# Patient Characteristics and Different Decision Paths for Establishing Palliative Care for Patients Admitted via the Emergency Department

**DOI:** 10.3390/jcm14248935

**Published:** 2025-12-18

**Authors:** Christiane Munsch, Sebastian Bergrath, Jessika Stefanie Kreß, Ullrich Graeven, Jana Vienna Rödler

**Affiliations:** 1 Palliative Care Section, Kliniken Maria Hilf Moenchengladbach, Academic Teaching Hospital of RWTH Aachen University, 41063 Moenchengladbach, Germany; 2 Medical Faculty, RWTH Aachen University, 52074 Aachen, Germany; sebastian.bergrath@mariahilf.de; 3 Center of Clinical Acute and Emergency Medicine, Kliniken Maria Hilf Moenchengladbach, Academic Teaching Hospital of RWTH Aachen University, 41063 Moenchengladbach, Germany; j.kress@asklepios.com (J.S.K.); janavienna.roedler@mariahilf.de (J.V.R.); 4 Department of Hematology, Oncology and Gastroenterology, Kliniken Maria Hilf Moenchengladbach, Academic Teaching Hospital of RWTH Aachen University, 41063 Moenchengladbach, Germany; ullrich.graeven@mariahilf.de

**Keywords:** palliative care, emergency department, symptom burden, early integration, staff education, screening tools

## Abstract

**Background:** Up to 10% of emergency department (ED) patients present with palliative care needs. Despite rising demand for palliative expertise in acute care, ED processes for these patients remain heterogeneous, and data from German EDs are limited. **Methods:** This retrospective cohort study included all patients presenting to the ED of a 754-bed academic hospital in 2023 who were later admitted to the palliative care ward. Demographics, symptom burden at ED and palliative care admission, length of stay (LOS), and discharge outcomes were analyzed after ethics approval (EK 24-062). Patients transferred to palliative care later during hospitalization (group 1) were compared with those directly transferred after ED treatment (group 2). **Results:** Among 229 included patients, 190 were identified as group 1 and 39 as group 2 patients. Demographics and cancer prevalence were comparable (68.4% vs. 69.2%). In group 1, fatigue, neurological symptoms, dyspnea, and anxiety/restlessness significantly increased during hospitalization, while anxiety/restlessness decreased significantly in group 2. Before palliative admission, LOS in group 1 was 15.2 ± 13.9 days; 36.8% required intensive or stroke unit care. LOS in the palliative ward (8.0 ± 6.6 vs. 9.3 ± 10.3 days, *p* = 0.45) and discharge alive rates (27.1% both groups) did not differ. **Conclusions:** Early recognition and management of palliative needs in the ED may reduce symptom burden. Once specialized palliative care was established, LOS and mortality were comparable across groups, highlighting the value of standardized assessments for early identification and integration of palliative care in acute settings.

## 1. Introduction

Emergency departments (EDs) take care of all kinds of patients, including those injured or critically ill. This also includes patients with a palliative status, who account for a relevant share of up to 10% of all patients in EDs [1]. However, only limited clinical data on palliative patients in the ED setting from German-speaking countries is currently available [2,3]. Furthermore, when the ongoing demographic change is taken into consideration, the palliative care patient population is expected to grow significantly in the future [4,5].

In addition to demographic aging, advances in medical treatment—especially in oncology—lead to prolonged survival times, resulting in a growing number of patients living with severe chronic illnesses or progressive incurable diseases. These patients frequently experience complex symptom burdens such as dyspnea, pain, or severe infections, which often prompt presentation to the ED. At the same time, ED environments are traditionally oriented toward curative and acute interventions rather than symptom control, communication regarding goals of care, or end-of-life decision-making. This creates a clinical and organizational challenge when treating patients with palliative needs in this acute medical setting.

International studies have highlighted the importance of early identification of palliative care needs in the ED and emphasized the role of standardized criteria, such as the Supportive and Palliative Care Indicators Tool (SPICT), to support decision-making in acute care [6]. Nonetheless, little is known about how these criteria are applied in practice and how they relate to subsequent patient pathways—particularly regarding whether patients are transferred to intensive care units (ICUs), general wards, or palliative care units.

This study aims to determine the incidence and characteristics of palliative care patients admitted to the hospital via the ED. It further compares patients directly classified as palliative with those initially considered non-palliative but who later received specialized palliative care during hospitalization. Possible differences regarding symptom burden at admission, patient population (oncological vs. non-oncological), length of stay (LOS) in the palliative ward and number of patients that could be discharged alive should be evaluated between these different groups.

## 2. Materials and Methods

### 2.1. Study Design

This retrospective, exploratory cohort study analyzed the characteristics, incidence, and symptom burden of ED patients who were subsequently admitted to the palliative care ward between 1 January 2023, and 31 December 2023, and were ultimately discharged either alive or deceased.

### 2.2. Study Population

Patients were eligible for inclusion if they presented to the ED and were later admitted to the palliative care ward during the study period. Patients with a palliative status who died directly in the ED were excluded. Likewise, patients who could not be transferred to the palliative care unit after emergency treatment due to limited bed capacity were not included.

### 2.3. Group Classification

Two patient groups were defined based on their palliative status at the time of ED presentation:

**Group 1:** Patients initially treated as non-palliative but subsequently identified as requiring specialized palliative care.
**Group 2:** Patients identified as palliative at initial ED assessment and directly transferred to the palliative care ward following initiation of symptom control by ED staff.

Comparisons were made between these groups regarding patient characteristics, symptom burden at admission, LOS in the palliative care ward, and discharge status (alive vs. deceased).

### 2.4. Assessment of Palliative Care Need

To determine the necessity for specialized palliative care, the modified Supportive and Palliative Care Indicators Tool (SPICT) was applied [6]. The SPICT criteria were adapted to account for the limited availability of general indicators and specific diagnostic information in the emergency setting. At the time of admission to the ED, the SPICT criteria were applied in a simplified form, assessing only whether an advanced disease of any organ system was present. In addition, it was recorded whether a palliative status had already been established prior to admission, and the number of inpatient stays during the previous 12 months was determined. Further differentiation of disease stage or complexity was not possible retrospectively due to limited data availability.

### 2.5. Symptom and Pain Assessment

At ED admission, symptom assessment was limited to the presence or absence of symptoms (yes/no); the intensity or severity of symptoms was not measured. Upon admission to the palliative care ward, patients’ symptom burden was assessed using the standardized palliative care basic assessment, incorporating elements of the modified Minimal Documentation System (MIDOS) symptom score in accordance with the guidelines of the German Association for Palliative Medicine (Deutsche Gesellschaft für Palliativmedizin, DGP) [7,8]. The intensity of 15 predefined symptoms—nausea, vomiting, constipation, weakness, loss of appetite, fatigue/exhaustion, sleep disturbance, wound-related problems, dyspnea, edema, need for assistance with activities of daily living, depression/lack of motivation, anxiety, confusion/disorientation, care-related problems, and family overload—was rated on a four-point scale from none (0) to severe (3). Pain intensity was additionally documented using a numerical rating scale.

### 2.6. Local Setting

Our academic teaching hospital (Kliniken Maria Hilf Moenchengladbach, Moenchengladbach, Germany) comprises 754 beds including 45 ICU beds, an 18-bed supraregional stroke unit, a 12-bed palliative ward and a level II ED. The hospital is certified as oncologic center, cardiac arrest center and regional trauma center (level II). The core medical team of the ED consists of consultant physicians, specialists, and resident doctors in training. As of 2023, a 24/7 off duty specialized inpatient palliative care service has been established alongside the daytime.

### 2.7. Outcomes

All patients admitted to the palliative ward between 1 January 2023, and 31 December 2023, were included if the admission to the hospital occurred via the ED. We modified the published supportive SPICT criteria [6].

The following outcomes were analyzed:

Comparison of patient characteristics between patients admitted primarily curative (ED → Stroke/ICU/other ward → palliative ward = group 1) and those admitted primarily with a palliative status (ED → palliative ward = group 2):

○ Main symptoms at ED arrival;
○ Clinical indicators of one or multiple life-limiting conditions (cancer, dementia, frailty, neurological disease, heart/vascular disease, respiratory disease, kidney disease, liver disease, weight loss);
○ Differentiation between oncological vs. non-oncological patient population;
○ Previously known palliative status;
○ Number of hospital stays as in-patient in the last 12 months prior to admission;
○ Number of in-patient days in the last 12 months prior to admission;
○ Severity and number of symptoms (symptom burden) at admission to the palliative ward (standardized palliative care basic assessment with the modified minimal documentation system (MIDOS) symptom score) [8];
○ LOS in the palliative ward;
○ Proportion of patients discharged alive from palliative ward.

### 2.8. Data Sources

All data were extracted from medical routine data only. An electronic health record system (iMedOne, Deutsche Telekom Healthcare and Security Solutions GmbH, Bonn, Germany) served as the main data source. Additionally, all paper-based documents of the patient charts were screened. Study data were transferred into a secured and user-restricted Excel database (version 16.103.4, Microsoft Corp., Redmond, WA, USA) and was secondarily pseudonymized.

### 2.9. Ethics Approval and Consent to Participate

This retrospective study was conducted in accordance with the ethical principles of the Declaration of Helsinki. An ethics application was submitted to the responsible ethics committee (Medical Faculty of RWTH Aachen University, Germany) and approved on 13 February 2023 (registration number EK 24-062). Additionally, the study was registered at the Clinical Trial Center of RWTH Aachen University, Germany (CTC-A Number 24-074) prior to the start of the study. According to national regulations, obtaining informed consent from all participants was not required for this type of study. The need for informed consent was waived by the responsible ethics committee as part of the approval process.

### 2.10. Statistics

Descriptive data was displayed as mean and median with standard deviation (SD) and interquartile ranges (IQR). We compared admission characteristics between both groups using the Fisher’s exact test and Welch’s test where applicable. Due to exploratory character of the study, *p*-values < 0.05 were considered to be significant. All statistical analyses were carried out with Prism 8.4.2 GraphPad Software, San Diego, CA, USA.

## 3. Results

### 3.1. Demographic Baseline Data of the Study

During the study period, 38,395 cases were treated in the ED. Of these, 49% were treated as outpatient emergencies. A total of 422 patients were treated in the palliative care ward in the same period. Of these 422 patients, 239 (56.6%) were initially admitted to the hospital via the ED. Patients who could not be transferred to the palliative care ward at the time due to a lack of local capacity were not considered in this dataset (*n* = 10). Overall, 190 patients were primarily transferred from the ED to the ICU, stroke unit or normal ward in a non-palliative status (group 1). The 39 remaining primarily palliative patients were directly transferred to the palliative ward (group 2) after ED care.

The demographic baseline data of the collective is summarized in Table 1. There were no significant differences in the demographic data between both groups, except for the inpatient admission by private practice physicians.

### 3.2. Symptom Burden at ED Admission

Table 2 summarizes the symptoms complained by patients at initial contact in the ED. Group 2 differed from group 1 in that it had a higher number of patients complaining of neurological symptoms (*p* = 0.047), pain (*p* < 0.001), reduced vigilance (*p* = 0.03), anxiety/restlessness (*p* < 0.001) and nausea (*p* = 0002). No other significant differences in the symptom burden at initial contact in the ED were identified.

### 3.3. Modified SPICT Criteria at ED Admission

Table 3 presents the modified SPICT criteria clustered by the respective disease/organ system for the study collective. For the entire study collective (group 1, group 2), positive SPICT criteria were identified, apart from a single patient from group 1. On average, 3.4 positive SPICT criteria have been identified for group 1 (min. 0, max. 6, SD ± 1.4) as well as for group 2 (min. 1, max. 5, SD ± 1.0) at hospital admission, *p* = 1.0. The average number of inpatient stays in the 12 months preceding the current hospital admission was 1.7 in group 1 (min. 0, max. 9, SD ± 2) and 1.2 in group 2 (min. 0, max. 6, SD ± 1), *p* = 0.023. In the last 12 months prior the current admission patients of group 1 were hospitalized on average for 18.3 days (min. 0, max. 187, SD ± 24.3), versus 14.0 days for the patients of group 2 (min. 0, max. 53, SD ± 16.8), *p* = 0.184.

In group 1, 130/190 patients (68.4%) had a pre-existing oncological status while in group 2 the amount was 27/39 patients (69.2%), *p* = 0.912. Patients in group 1 had significantly more solid tumor disease without metastasis than those in group 2 (*n* = 37/190, 19.5% vs. *n* = 1/39, 2.6%), *p* = 0.01. A documented palliative status upon admission to the ED was present in 32.1% (61/190) of patients in group 1 and in 87.2% (34/39) of patients in group 2 (*p* < 0.001). Overall, 21.1% of patients in group 1 (40/190) had an oncological diagnosis as the admission diagnosis and 59.0% (23/39) in group 2, *p* < 0.001.

### 3.4. Symptom Burden at the Timepoint of ED Admission to the ED vs. Admission to the Palliative Ward

**Figure 1:**
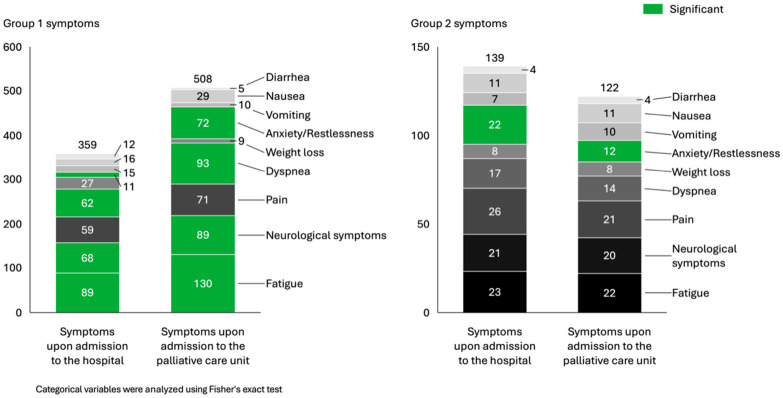
Number of patients reporting distressing symptoms between ED admission and admission to the palliative ward.

Figure 1 presents the number of patients complaining about distressing symptoms between the timepoint of ED admission and admission to the palliative ward. Weight loss was the only symptom where a significant decrease during hospitalization in group 1 (*p* = 0.0025) could be identified. The number of patients complaining about anxiety/restlessness, dyspnea, neurological symptoms and fatigue significantly increased during hospitalization (*p* < 0.05) in group 1 between the time point of ED care (non-palliative status) and admission to the palliative ward in palliative status. In contrast, group 2 displayed a significant decrease of the number of patients suffering from anxiety/restlessness (*p* = 0.04) after having started with symptom control in the ED by ED staff and being transferred to the palliative ward directly after that.

### 3.5. Admission Pathways and LOS

The LOS prior to admission to the palliative care ward (group 1) showed a mean of 15.2 ± 13.9 days (min 1, max 67 days). In group 1, 26.8% (51/190) of patients were transferred directly from the ICU or stroke unit to the palliative care ward. Of the 73.2% (139/190) of patients who were transferred from a normal ward to the palliative care ward, 13.7% (19/139) of patients had previously been in the ICU or the stroke unit. Group 1 showed an average LOS in the palliative care ward of 8.0 ± 6.6 days (min 0, max 39.0 days) while group 2 showed a period of 9.3 ± 10.3 days (min 1.0, max 49.0 days), *p* = 0.45.

### 3.6. Discharge Outcomes

Table 4 represents the type of discharge for group 1 and group 2 patients. Overall, 27.1% of patients were discharged alive from the palliative care ward with no significant difference between the two groups (*n* = 54/190; 28.42% vs. *n* = 8/39; 20.51%), *p* = 0.43. The care provided by general outpatient palliative care (AAPV) cannot be accurately evaluated due to the small number of cases, although it is statistically significant.

## 4. Discussion

In this study, we compared ED patients who were either primarily admitted to specialized palliative care or transferred there after a non-palliative treatment phase. The analysis focused on differences in symptom burden at presentation, timing and mode of palliative care initiation, underlying disease type (oncological vs. non-oncological), and hospital outcomes. Both groups exhibited comparable demographics and admission routes to the ED. Symptom burden on ED admission was largely similar, except for higher levels of neurological symptoms, pain, reduced vigilance, anxiety/restlessness and nausea in primarily palliative patients. A pre-existing palliative status was documented in 32.1% of group 1 and 87.2% of group 2, while an oncological disease as admission diagnosis was more frequent among primarily palliative patients (21.1% vs. 59.0%). LOS and discharge rates from the palliative ward were comparable, with approximately 27% of patients discharged alive. Importantly, we found no evidence that primary transfer to the palliative care ward was influenced by triage decisions related to limited ICU bed availability.

Although the overall symptom burden at ED admission was similar between the two groups, primarily palliative patients reported higher levels of neurological symptoms, pain, reduced vigilance, anxiety/restlessness and nausea, which may have been the reason for their presentation to the ED. This observation is consistent with Böhm et al., who found that EDs frequently serve as first contact points for palliative patients experiencing symptom exacerbation or caregiver overload [9]. Early initiation of symptom control by the ED staff and direct transfer to a palliative care ward may reduce distress, particularly regarding anxiety and restlessness. Importantly, adopting a primary palliative concept in the ED did not increase mortality. These results align with previous evidence from both emergency-based and general palliative care research. In a randomized clinical trial across 29 EDs in the United States, Grudzen et al. demonstrated that ED–initiated palliative care improved quality of life in patients with advanced cancer without shortening survival [10]. Beyond the emergency setting, several population-based studies have demonstrated consistent findings indicating that palliative care initiated in the ED or in a timely manner overall improves quality of life without shortening survival, thereby supporting the international trend toward early integration of palliative principles within acute and emergency care settings [11,12,13,14,15,16].

Weight loss was the only symptom showing a statistically significant clinical improvement during hospitalization in group 1 (*p* = 0.0025), likely reflecting better hydration, and nutrition. However, documentation bias cannot be excluded, as weight loss was often based on retrospective or subjective assessments.

In group 1, anxiety/restlessness, dyspnea, neurological symptoms, and fatigue differed significantly between the ED care phase and the subsequent palliative admission. For secondarily palliative patients, symptom escalation may have led to the decision of transition from curative to specialized palliative care. Furthermore, the high proportion of non-oncological patients in group 1 suggests frequent palliative needs in medical wards who are possibly less familiar with palliative principles. Given that many hospitalized patients with life-limiting illnesses have unrecognized palliative needs, this highlights the importance of establishing clear clinical guidelines and implementing systematic screening [14,17]. In this context, the systematic review by El Mokhallati et al. identified 25 studies that examined the implementation of screening tools designed to detect palliative care needs [17]. However, while an so-called S3 guideline exists for incurable cancer, no equivalent guideline currently addresses non-oncological patients [18]. Given that 31% of our cohort were non-oncological, this represents a significant gap. Despite comparable needs, these patients remain underrepresented in specialized palliative care [19]. Since only one in six Europeans die from cancer, most of the end-of-life and palliative emergency cases fall outside current guideline coverage [20]. Publications such as Michels et al. provide essential groundwork for addressing this gap [21]. For the first time, these consensus guidelines signify a nationwide commitment to systematically integrating palliative care into ICU and ED practice for patients with non-oncological conditions. In our study, a pre-existing palliative status was a key factor influencing direct transfer to the palliative unit (32.1% vs. 87.2%). Given that 99.6% of all patients met positive SPICT criteria, complementing these criteria with the use of a standardized screening tool could help to systematically and more promptly identify palliative care needs, even before a formal palliative status is established [22,23,24]. In line with findings from other investigations, the first German studies by Schmitz et al. demonstrated that, beyond the use of positive SPICT criteria, the Palliative Care and Rapid Emergency Screening (P-CaRES) tool can serve as a feasible and reliable instrument for identifying palliative care needs in the ED, exhibiting high face, content, and construct validity [23].

Furthermore, Fidelsberger et al. were the first to apply the P-CaRES tool to identify palliative care needs among patients admitted to an internal medicine ward. Their study demonstrated that the tool can be effectively utilized in the inpatient context to systematically detect patients requiring palliative care support [25].

Integrating patients into specialized palliative teams can prevent further symptom escalation and unnecessary hospital days. Evidence consistently shows that inpatient palliative care improves satisfaction, aligns care with patient goals, and reduces intervention rates [19,26,27]. Systematic reviews have demonstrated no increase in LOS or mortality once specialized palliative care is established [28,29]. These observations are corroborated by our data, which, as noted earlier, indicate that the timing of specialized palliative care initiation did not influence the length of stay in the palliative ward or mortality across both groups.

International guidelines emphasize structured communication, symptom management, and decision-making support, including palliative training for ICU and ED physicians [30,31,32]. In our cohort, over one-third of group 1 patients had ICU or stroke unit stays, highlighting this need. Previous studies have shown that up to 75% of ICU patients have palliative needs yet often lack corresponding support [33,34,35]. As an example, with regard to the previously mentioned importance of implementing dedicated screening tools, Spadaro et al. applied the NECesidades PALiativas (NECPAL) instrument as a screening method and found that approximately one-third of all intensive care admissions during the study period met criteria indicating a need for palliative care [34]. The ongoing “Enhancing Palliative Care in Intensive Care Units” (EPIC) project aims to improve early palliative integration in ICUs through structured training, screening, and teleconsultation [36]. The study consists of three main phases. In the first phase, ICU staff will receive training in palliative care principles and communication. In the second phase, checklists for the early identification of patients with potential palliative care needs will be developed and implemented. In the third phase, interdisciplinary consultations by specialized palliative care professionals will be introduced to provide team-based palliative support and guidance. Similar approaches are warranted in EDs, encompassing the adoption of palliative principles, interdisciplinary collaboration, and targeted research to better define patient needs and outcomes [21,37,38]. This need is emphasized by observations from Rose et al., who reported that acutely dying patients are treated in the ED approximately once per week in an ED treating 40,000 patients/a [1]. Furthermore, studies revealed inconsistent ED access and limited palliative expertise, underscoring areas for improvement [1,9,35].

Finally, the projected 25% increase in deaths in Germany by 2050 highlights the growing importance of palliative services in an aging population [5]. As care demand evolves with demographics, clinical standards, and policy priorities, continuous adaptation of palliative structures within EDs and hospitals will be essential to ensure equitable, high-quality end-of-life care.

### Limitations

This study has several limitations. First, the retrospective design of this study limits the ability to establish causal relationships and is subject to potential biases inherent in secondary data analysis, such as incomplete documentation and variability in data recording.

Another major limitation of this study is the non-standardized symptom assessment upon hospital admission in the ED. It was only checked whether symptoms were present at the time of admission or not. In contrast, the symptom burden was recorded on admission to the palliative care unit using a standardized palliative care baseline assessment and the modified MIDOS symptom score [8].

The number of cases in this study can still be considered limited and in need of expansion. Furthermore, the study only considered patients from a single hospital. For improved validity, a multicenter study with a larger patient population should be conducted in future.

Due to the lack of data for relevant criteria upon arrival in the ED, a modification of the SPICT criteria had to be made. Therefore, diagnoses were not differentiated further. Due to this circumstance, the findings of this study might be slightly distorted by this lack of information.

## 5. Conclusions

The early recognition and management of palliative needs in the ED is crucial to reducing symptom burden. Once specialized palliative care was established, LOS and discharge rates were similar across groups. These findings emphasize the importance of standardized, repeated symptom assessments, early referral to specialized palliative care, and improved training of ED and ICU staff, particularly for non-oncological patients, to optimize care and resource use.

## Figures and Tables

**Table 1:** Demographic baseline data of the study.

Demographics				
Parameter	∑	Group 1	Group 2	*p*-Value
*n*	229	190	39	
gender distribution m/f/d	111 (48.5%)/118 (51.5%)/0 (0%)	94 (49.5%)/96 (50.5%)/0 (0%)	17 (43.6%)/22 (56.4%)/0 (0%)	0.60
age in years (M ± SD)	75 ± 12	75 ± 12	75 ± 12	1.00
average weight in kg (min/max)	67 (35/157)	68 (35/157)	66 (39/100)	0.49
average height in cm (min/max)	1.70 (1.50/1.95)	1.69 (1.50/1.95)	1.70 (1.55/1.86)	0.53
mean BMI (min/max)	23 (14/46)	24 (14/46)	23 (14/39)	0.34
type of hospital admission				
emergency medical service	137 (60.1%)	116 (61.1%)	21 (55.3%)	0.47
independent	39 (17.1%)	35 (18.4%)	4 (10.5%)	0.25
hospital admission by an office-based physician	36 (15.8%)	24 (12.6%)	12 (31.6%)	**0.01**
hospital-based outpatient department	7 (3.1%)	7 (3.7%)	0 (0.0%)	0.61
others	10 (4.4%)	8 (4.2%)	2 (5.3%)	0.68

Legend: m, male; f, female; d, diverse; M, mean; SD, standard deviation; BMI, Body Mass Index. Categorical variables were analyzed using Welch´s test and Fisher´s exact test. Bold *p*-values indicate statistically significant results (*p* < 0.05).

**Table 2:** Number of patients complaining about distressing symptoms at first contact in the ED.

Symptoms at First Contact in the ED				
	∑	Group 1	Group 2	*p*-Value
fatigue (*n*)	112	89	23	0.23
%	48.9%	46.8%	59.0%	
neurological symptoms (*n*)	89	68	21	**0.047**
%	38.9%	35.8%	53.8%	
pain (*n*)	85	59	26	**<0.001**
%	37.1%	31.1%	66.7%	
dyspnea (*n*)	79	62	17	0.2
%	34.5%	32.6%	43.6%	
reduced vigilance (*n*)	57	42	15	**0.03**
%	25.8%	23.1%	38.5%	
weight loss (*n*)	35	27	8	0.32
%	15.3%	14.2%	20.5%	
anxiety/restlessness (*n*)	33	11	22	**<0.001**
%	14.4%	5.8%	56.4%	
vomiting (*n*)	22	15	7	0.06
%	9.6%	7.9%	17.9%	
nausea (*n*)	27	16	11	**0.002**
%	11.8%	8.4%	28.2%	
diarrhea (*n*)	16	12	4	0.49
%	7.0%	6.3%	10.3%	

Categorical variables were analyzed using Fisher´s exact test. Bold *p*-values indicate statistically significant results (*p* < 0.05).

**Table 3:** Modified SPICT Criteria at ED Admission.

Modified SPICT Criteria				
	∑	Group 1	Group 2	*p*-Value
other diseases (*n*)	184	152	32	1.00
%	80.3%	80.0%	82.1%	
cardiovascular diseases (*n*)	180	148	32	0.67
%	78.6%	77.9%	82.1%	
kidney diseases (*n*)	71	58	13	0.71
%	31.0%	30.5%	33.3%	
diseases of the nervous system (*n*)	68	58	10	0.70
%	29.7%	30.5%	25.6%	
cancer with metastasis (*n*)	119	93	26	0.05
%	52.0%	48.9%	66.7%	
cancer without metastasis (*n*)	38	37	1	**0.01**
%	16.6%	19.5%	2.6%	
lung diseases (*n*)	56	49	7	0.41
%	24.5%	25.8%	17.9%	
dementia/frailty (*n*)	24	18	6	0.26
%	10.5%	9.5%	15.4%	
liver diseases (*n*)	12	9	3	0.43
%	5.2%	4.7%	7.7%	
palliative status				
pre-existing palliative status (*n*)	95	61	34	**<0.001**
%	41.5%	32.1%	87.2%	
connection to AAPV/SAPV (*n*)	20	14	6	0.12
%	8.7%	7.4%	15.4%	
inpatient stays in the last 12 months				
number of patients with an inpatient stay in the last 12 month (*n*)	152	128	24	0.58
%	66.4%	67.4%	61.5%	

Legend: SPICT, supportive and palliative care indicator tool; AAPV, general ambulatory outpatient palliative care; SAPV, specialized ambulatory palliative care. Categorical variables were analyzed using Fisher´s exact test. Bold *p*-values indicate statistically significant results (*p* < 0.05).

**Table 4:** Type of discharge of the study cohort.

Discharge Status				
	∑	Group 1	Group 2	*p*-Value
death (*n*)	167	136	31	0.43
%	73.2%	71.6%	79.5%	
AAPV (*n*)	5	2	3	**0.04**
%	2.2%	1.1%	7.7%	
SAPV (*n*)	16	16	0	0.07
%	7.0%	8.4%	0.0%	
hospice (*n*)	41	36	5	0.48
%	18.0%	18.9%	12.8%	

Legend: AAPV, general ambulatory outpatient palliative care; SAPV, specialized ambulatory palliative care. Categorical variables were analyzed using Fisher´s exact test. Bold *p*-values indicate statistically significant results (*p* < 0.05).

## Data Availability

The datasets used and/or analyzed during the current study are available from the corresponding author on reasonable request.

## Abbreviations

The following abbreviations are used in this manuscript:

AAPV	Allgemeine Ambulante Palliativversorgung (general outpatient palliative care)
ED	Emergency Department
EPIC	Enhancing Palliative Care in Intensive Care Units
ICU	Intensive Care Unit
IQR	Interquartile Ranges
LOS	Length of stay
MIDOS	Minimal Documentation System
NECPAL	NECesidades PALiativas
P-CaRES	Palliative Care and Rapid Emergency Screening Tool
SAPV	Spezialisierte Ambulante Palliativ Versorgung (specialized outpatient palliative care)
SD	Standard Deviation
SPICT	Supportive and Palliative Care Indicators Tool
